# *Drosophila* female reproductive tract gene expression reveals coordinated mating responses and rapidly evolving tissue-specific genes

**DOI:** 10.1093/g3journal/jkab020

**Published:** 2021-01-28

**Authors:** Caitlin E McDonough-Goldstein, Kirill Borziak, Scott Pitnick, Steve Dorus

**Affiliations:** Center for Reproductive Evolution, Biology Department, Syracuse University, Syracuse, NY, USA

**Keywords:** reproduction, sexual selection, ejaculate–female interactions, fertility

## Abstract

Sexual reproduction in internally fertilizing species requires complex coordination between female and male reproductive systems and among the diverse tissues of the female reproductive tract (FRT). Here, we report a comprehensive, tissue-specific investigation of *Drosophila melanogaster* FRT gene expression before and after mating. We identified expression profiles that distinguished each tissue, including major differences between tissues with glandular or primarily nonglandular epithelium. All tissues were enriched for distinct sets of genes possessing secretion signals that exhibited accelerated evolution, as might be expected for genes participating in molecular interactions between the sexes within the FRT extracellular environment. Despite robust transcriptional differences between tissues, postmating responses were dominated by coordinated transient changes indicative of an integrated systems-level functional response. This comprehensive characterization of gene expression throughout the FRT identifies putative female contributions to postcopulatory events critical to reproduction and potentially reproductive isolation, as well as the putative targets of sexual selection and conflict.

## Introduction

The contribution of the female reproductive tract (FRT) to reproductive success was unequivocally demonstrated in 1951 with the observation that capacitation (*i.e.*, sperm acquiring the capacity to fertilize) is dependent on sperm interactions with the FRT (Austin 1951; Chang 1951). Over the ensuing decades, FRT morphology, physiology, and secretions have increasingly been shown to support sperm motility and viability as well as influence both intraspecific variation in reproductive outcomes (Pitnick *et al.* 2009; Wolfner 2011; Orr and Brennan 2015; Suarez 2016) and reproductive isolation between species (Howard *et al.* 2009; McDonough *et al.* 2016). However, a refined understanding of the genetic basis of many female-mediated mechanisms critical to fertility remains enigmatic.

Investigations of specific FRT tissues suggest that the function of this system depends upon the coordination of its composite parts. In mice, for example, knockout of endometrial glands prevents the establishment of pregnancy because the luminal epithelium of the FRT does not undergo the molecular and structural changes necessary to support blastocyst implantation (Kelleher *et al.* 2016, 2018). In the fruit fly, *Drosophila melanogaster*, the loss of FRT glandular tissues also results in reduced fertility due to defects in the functions associated with other FRT tissues, including the seminal receptacle (*i.e.*, sperm motility dysfunction), the bursa (*i.e.*, delayed oviposition) and the ovary/oviduct (*i.e.*, delayed ovulation) (Anderson 1945; Allen and Spradling 2007; Schnakenberg *et al.* 2011; Sun and Spradling 2013). However, even in this model system, the contributions of the discrete tissues to the extracellular FRT environment, which female gene products interacts with the ejaculate, and the integrated functionality of the FRT have yet to be well characterized.

**Figure 1:**
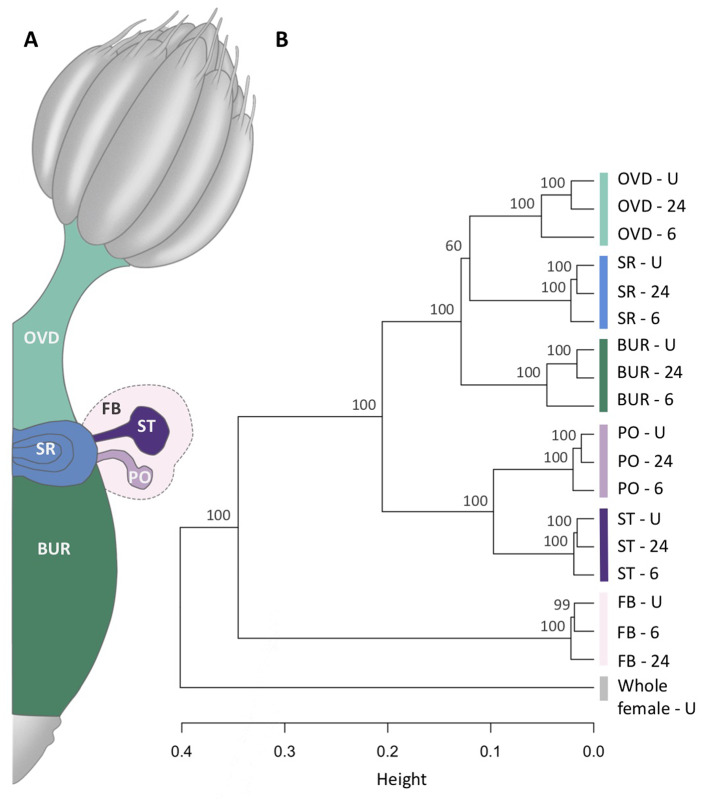
FRT gene expression. (A) The FRT of *Drosophila melanogaster* consists of five tissues: the epithelial tissues of the bursa (BUR), oviduct (OVD), seminal receptacle (SR), and glandular tissues spermathecae (ST), and parovaria (PO). The spermathecae and parovaria are surrounded by an FRT-associated fat body (FB). Note, the seminal receptacle is on the ventral side of the FRT at the approximate junction of the bursa and oviduct whereas the spermathecae and parovaria arise from the dorsal side. (B) Hierarchical clustering of average gene expression for each tissue at three time points: unmated, 6 hs postmating, and 24 h postmating. In addition to FRT tissues and associated fat body, we analyzed unmated whole female which included the ovaries but excluded the lower FRT. Clustering was based on average correlation distance and approximately unbiased *P*-values (%) are presented for each node. FRT tissues formed two primary groups that distinguished epithelial and glandular tissues. It was noteworthy that within all FRT tissues, but not the FRT-associated fat body, there was a consistent clustering of unmated and 24 h postmating samples with 6 h postmating as the outlier.

Insects are a tractable system for exploring the molecular complexity of FRT tissues as they exhibit morphological and functional compartmentalization of tissue cell types (Gillott 2003). Over a century of research with *D. melanogaster* has established the morphological, histological, developmental, functional and, to a more limited extent, molecular properties of individual FRT tissues. The *D. melanogaster* FRT consists of five tissues: the bursa copulatrix, oviduct, seminal receptacle, spermathecae, and parovaria (Figure 1A; Nonidez 1920). The bursa copulatrix (bursa or “uterus”) is the site of insemination, fertilization, and embryo retention until oviposition. The oviduct regulates ovulation and egg activation (Heifetz *et al.* 2001; Mattei *et al.* 2015). There are two specialized sperm-storage organs: the seminal receptacle and a pair of spermathecae. The seminal receptacle is a muscular tube that is the primary source of sperm for fertilization (Manier *et al.* 2010). The spermathecae are sclerotized capsules surrounded by secretory cells which are necessary for maintenance of sperm viability in both tissues (Anderson 1945; Allen and Spradling 2007; Schnakenberg *et al.* 2011; Sun and Spradling 2013). The parovaria (or female accessory glands) consist of secretory cells morphologically and developmentally similar to the spermathecae (Sun and Spradling 2012). The parovaria have been the focus of limited investigation, but may possess redundant functions with the spermathecae in supporting sperm storage and ovulation (Anderson 1945; Sun and Spradling 2012).

Despite the diversity in FRT morphology across the animal kingdom, there is substantial congruence in the critical postmating reproductive events that occur within the FRT environment, including the receipt and processing of the ejaculate, sperm movement and storage, ovulation, egg activation, and fertilization. In *D. melanogaster* the timeline of these postmating reproductive events has been divided into three temporal phases following copulation (Carmel *et al.* 2016). Phase 1 (lasting approximately 6 h) includes ejaculate transfer, formation and ejection of the ejaculate plug (along with excess and displaced sperm), sperm migration to the storage organs, and initiation of ovulation and oviposition. During this phase, the FRT also undergoes conformational changes and tissue remodeling including increased secretory capacity (Adams and Wolfner 2007; Kapelnikov *et al.* 2008a). Phase 2 (6–24 h) includes the maintenance of stored sperm and elevated rates of ovulation and oviposition. Finally, during phase 3 (>24 h), females are in a relatively constant state of egg laying and become increasingly receptive to remating.

The male stimuli that induce female postmating responses, including changes in gene expression, have been the subject of intense genetic and molecular investigation. These stimuli include courtship and copulation behaviors as well as the effects of sperm and seminal fluid proteins (SFPs), which induce changes in female behavior, metabolism, immune function and reproductive physiology (Wolfner 2002). Far less is known about how these male stimuli are received [with the exception of one known SFP receptor (Yapici *et al.* 2008)] or the mechanisms that govern postmating responses. Nevertheless, neuromodulators appear to play an important role in both temporally coordinated changes across the FRT and the establishment of distinct regional responses (Heifetz *et al.* 2014; Carmel *et al.* 2016).

To advance our understanding of FRT function, we conducted a systematic parallel analysis of all FRT tissues—including the first expression analysis of the parovaria, bursa, and FRT-associated fat body. Specifically, we aimed to determine (1) whether tissues make distinct contributions to the FRT that may form the basis of regional microenvironments, (2) whether genes of the FRT experience accelerated evolution, and (3) the extent to which tissues of the FRT exhibit temporally and functionally coordinated molecular responses to mating. Our analyses reveal tissue-specific patterns of expression and system-level responses of the FRT to mating that are indicative of integrated functionality across the FRT.

## Materials and methods

### Fly maintenance and mating

*D. melanogaster* wild-type lab strain LH_M_ was reared in standard conditions at room temperature (∼23^°^C) with a natural light cycle on a yeast, cornmeal, agar, and molasses media. Within 14 h of eclosion, flies were anesthetized with CO_2_, separated by sex, and matured in single-sex vials of approximately 10 flies with 1.5 cm^2^ of media supplemented with live yeast for 3–8 days (ages were equivalently distributed across samples; mode 6 days). Before use, vials containing females were examined for larvae to confirm unmated status. Matings were conducted in female vials with the addition of approximately 15 males aged 3–8 days (ages randomly distributed across matings). Dissections were conducted on unmated females and at 6 h postmating (±1 h) or 24 h postmating (±2 h) following time of pairing.

### Tissue dissection and RNA isolation

Tissue dissections occurred across collections of approximately 50–75 females and were then pooled to obtain samples with tissues from approximately 300 females. For each collection, females were etherized and their FRT (including all lower somatic reproductive tissues), were isolated in 1× phosphate-buffered saline (PBS) with care to avoid contamination from the ovary, ovulated eggs, or nearby gut tissue. FRT tissues were then micro-dissected separating the bursa, oviduct (including as much of the lateral oviducts as possible), seminal receptacle, both spermathecae (including the duct when possible), both parovaria (including the duct when possible), as well as the FRT-associated fat body. Postmating FRT tissues were only collected if sperm was visible in the seminal receptacle, confirming that the female had successfully mated. Tissues were combined by tissue type, rinsed and then transferred directly into Trizol (ThermoFisher) using a capillary tube (Stripper Tips; Orgio). Two samples of 25 unmated whole females excluding the FRT were also collected. All collections were stored at −80°C. Samples were then pooled and homogenized with a pellet pestle and RNA was isolated using a phenol-chloroform extraction with phase-lock gels (ThermoFisher) and an overnight precipitation in isoproponal and 3M sodium acetate with 1.5 μl glycogen (Life Technologies) at −18°C to maximize RNA recovery. The precipitation was followed by DNAse I (LifeTechnologies) treatment for 30 min at 37°C and a second RNA isolation. RNA was re-suspended in RNase-free H_2_O and stored at −80°C.

### RNA-seq

Library preparation and 50 bp single-end sequencing was conducted at the Beijing Genomics Institute (BGI) on a BGIseq-500. Samples were analyzed in two batches to obtain a median of four biological replicates (and a minimum of two biological replicates) for each tissue and time point. Raw RNAseq data were processed by BGI to remove adapters and exclude reads with >10% unknown bases or >50% low-quality bases. The resultant high-quality reads were mapped to the FlyBase *D. melanogaster* reference genome (r6.21) (Thurmond *et al.* 2019) using hisat 2.1.0 with default settings plus the SAM option to suppress reads that do not align or represent novel splice sites (Kim *et al.* 2015). Read counts per gene were generated using StringTie (Pertea *et al.* 2015, 2016). All samples had >90% of reads map to unique genomic positions and a minimum of 12 million mapped reads (average of 24.7 ± 0.3 million mapped reads) (Supplementary Figure S1A). Genes were included in subsequent analysis if they had a counts per million (CPM) >2 in at least two replicates of a given tissue and time point. This reduced the data set from 17,752 genes to 8337 genes (Supplementary Figure S1, B and C). Nearly all genes (99.95%) identified as FRT-expressed were present in one or more unmated tissues. Read counts were TMM normalized using edgeR (Supplementary Figure S1D) (Robinson *et al.* 2010; McCarthy *et al.* 2012). Sample quality was checked by examining sample clustering with multidimensional scaling plots (Supplementary Figure S1, E and F) and Pearson’s’ correlations between samples with complete linkage hierarchical clustering (Supplementary Figure S1G). Data were highly reproducible with average *R*^2^ = 0.96 between replicates and the robust clustering of replicate samples and separation of tissues reflects the quality of isolated tissues samples.

### Transcriptome variation characterization and comparison

Relationships among samples were determined using two methods. First, hierarchical clustering using average linkage and correlation distance was performed on the average of log2 normalized CPM values for each tissue and time point to determine the approximately unbiased *P*-value confidence for each cluster (Suzuki and Shimodaira 2015). Second, principle components analysis was performed on normalized CPM values of unmated FRT samples. Loadings for the variables on the principle components were compared to gene expression differences by linear regression. FRT tissue data was also compared to FlyAtlas2 average FPKM (fragments per kilobase per million) including comparable samples of spermathecae and tissues of interest for comparison such as a general fat body sample (Leader *et al.* 2018). To compare our data to the FlyAtlas2 data we used hierarchical clustering with average linkage and correlation distance on scaled and centered expression for genes expressed in the FRT of all samples. We also examined the differences in gene expression among fat body samples with a linear regression.

### Differential expression

Differential expression analysis was used to identify genes that had significantly higher expression in unmated FRT tissues compared to the whole female as well as to identify genes that are differentially expressed between unmated and postmating time points. These analysis were performed with edgeR and conducted on each tissue separately with TMM normalization to account for batch effects (Robinson *et al.* 2010; McCarthy *et al.* 2012). Genes were considered significantly tissue-biased compared to the whole female with a stringent cutoff of Bonferroni corrected *P*-value ≤ 0.001 and log2FC ≥2. Genes were considered differentially expressed postmating if the Bonferroni corrected *P*-value was ≤0.01. Postmating differentially expressed genes were classified as “persistent” if they were differentially expressed in the same direction at both 6 and 24 h or were only differentially expressed only at 24 h relative to unmated samples. All other differentially expressed genes were considered “transient” (*i.e.*, not differentially expressed in the same direction at 6 and 24 h postmating).

### Tissue-specific expression

To measure tissue specificity of expression, we calculated τ on the unmated average expression of all tissues and the whole female (Yanai *et al.* 2005; Larracuente *et al.* 2008). τ measures tissue specificity on a scale from 0 (indicating broad expression across all tissues) to 1 (indicating expression in only one tissue). We used a cut off of τ > 0.9 to identify tissue-specific genes. As expected, the vast majority of tissue-specific genes (97%) were also amongst those identified as tissue-biased. The use of whole female in this analysis, as a representative for expression in other tissues throughout the body, led to the identification of genes that have a τ > 0.9 and maximum expression in the whole female. These genes were not included in subsequent analysis of FRT tissue-specific genes. We further used τ as a categorical variable in particular analysis, with five ranges of tau: (1) 0.0–0.5, (2) 0.5–0.7, (3) 0.7–0.8, (4) 0.8–0.9, and (5) 0.9–1. τ categories had an average of 1667 ± 285 genes, and 247  ± 58 genes with a signal annotation.

### Comparison of postmating time points

Gene expression changes between tissues and time points were compared by linear regressions. Fuzzy clustering was used to identify prominent patterns of gene expression change across time points in each tissue (Futschik and Carlisle 2005). Optimal cluster number was determined as the number prior to the largest decrease in minimal centroid distance. Cluster membership was determined as those genes with >0.75 membership values for those tissues with two clusters and >0.5 for those tissues with three clusters.

### Gene ontology and functional annotation

Gene ontology (GO) enrichments of tissue-specific or differentially expressed genes were determined using DAVID (Huang *et al.* 2009a, 2009b). For tissue-specific genes and genes with tissue-biased expression, we used a background of all FRT-expressed genes. For genes differentially expressed postmating in a given tissue, we used a background of all genes expressed in that tissue. Ontological categories were considered enriched if the Benjamini–Hochberg corrected *P *<* *0.05. Secretion signal annotation was determined from UniProt (The UniProt Consortium 2019).

### Molecular evolution

Orthology between *D. melanogaster* and *D. yakuba* (FB2017_02) (Thurmond *et al.* 2019) was established using a local installation of OrthoDB (Kriventseva *et al.* 2015) with default parameters. In cases where recent, lineage-specific paralogs were present in an orthology group, the longest coding sequence was used. Protein sequences were aligned using the linsi algorithm of MAFFT (Katoh and Standley 2013) and reverse translated. Evolutionary rates (dN/dS; ω) were estimated using the Goldman and Yang method (Goldman and Yang 1994) as implemented by PAML (Yang 2007). A small minority of genes with inflated divergence estimates were excluded due to likely misalignment.

### Statistical analysis and gene expression visualization

We used a chi-square test to analyze differences in number of genes (1) biased within or specific to each tissue, (2) differentially expressed across tissues, (3) differentially expressed at postmating times within each tissue, and (4) up or down regulated within each time and each tissue. We used a Kologmorov–Smirnoff test to compare distributions of genes with or without a signal annotation across τ. The effect of τ category and secretion as well as tissue-specific expression and secretion on evolutionary rate was analyzed using a nonparametric Kruskal–Wallis (on both the factors and the interaction between factors) with a Bonferroni correction for multiple of comparisons. Mean and 95% confidence intervals of evolutionary rates were determined with a bootstrap analysis. Expression patterns of tissue-specific genes were visualized using a heatmap with complete linkage and Euclidean distance hierarchical clustering for both genes and samples. Intersections between gene sets were visualized with UpsetR. Analysis was performed in R version 3.4.4 (R Core Team 2019).

### Data availability

Raw reads are available at the NCBI Gene Omnibus Project (GEO) accession GSE143759. Average expression and analyzed data can be found in Supplementary Table S1. Code and pre-processed data files for analysis are available on GitHub (github.com/CEMcDonoughGoldstein/FRT.Tissue.Transcriptomics). Supplementary results, figures, and tables are on figshare: https://doi.org/10.25387/g3.13503336.

## Results

We investigated the spatial and temporal transcriptional relationships among all five FRT tissues (bursa, oviduct and seminal receptacle, spermathecae, and parovaria; Figure 1A), as well as the FRT-associated fat body at three time points representing the phases of female postmating response (*i.e.*, unmated, 6 h postmating, and 24 h postmating). Expression profiles were highly reproducible across replicates for both tissues and timepoints (Supplementary Figure S1) and were also consistent with previous studies of *Drosophila* tissue expression (Supplementary Figure S2). Using hierarchical clustering analysis of average gene expression (Figure 1B), we found, first, that the five FRT tissues formed a single group which was distinct from both the FRT-associated fat body and the whole female (which included the ovaries but excluded the lower FRT). Second, there was high-confidence support for a division between tissues with glandular (spermathecae and parovaria) and primarily nonglandular epithelium [henceforth referred to as epithelial; bursa, oviduct and seminal receptacle; approximately unbiased (AU) *P *=* *1.00]. Third, that the three timepoints from each tissue formed distinct groups (AU *P *>* *0.6 for all). Finally, in all tissues, except the FRT-associated fat body, the 6 h postmating time point was consistently an outgroup to the more similar unmated and 24 h postmating time points (AU *P *=* *1.00 for all). In the following sections, we explore the genes that contribute to the observed variation across tissues.

### Tissue-biased genes were common to histologically similar tissues

**Figure 2:**
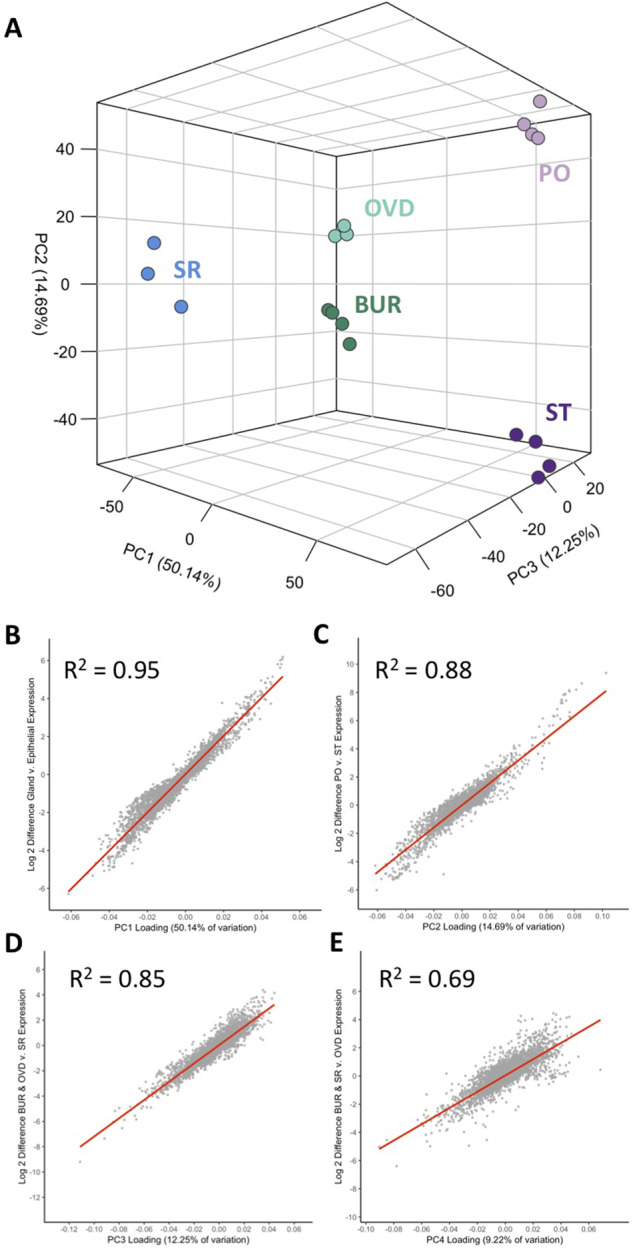
Expression variation across FRT tissues. (A) Principle component analysis of unmated FRT tissues identified three principle components that described 77.1% of the total variance and resolve FRT tissue transcriptomes into distinct clusters. The relationship of principle component rotation values to log2-fold differences in gene expression among tissue types was analyzed by linear regression in (B–E). (B) The first principle component was significantly correlated (*R*^2^ = 0.95) with the difference between glandular and epithelial tissue expression levels. (C) The second principle component was significantly correlated (*R*^2^ = 0.88) with the difference between spermathecae (ST) and parovaria (PO) expression levels. (D) The third and (E) the fourth principle components were significantly correlated to differences between epithelial tissues (*i.e.*, the seminal receptacle (SR) compared to the bursa (BUR) and oviduct (OVD), *R*^2^ = 0.85 and the oviduct compared to the seminal receptacle and bursa, *R*^2^ = 0.69, respectively).

We examined transcriptome variation among unmated FRT tissues using a principal component analysis. This analysis revealed four principal components that collectively explained 86.3% of transcriptome variation (Figure 2A). The first principal component captured 50.1% of the variation and was highly correlated with the difference in expression between epithelial and glandular tissues (*R*^2^ = 0.95, *P *<* *0.001; Figure 2B). The second principal component (14.69% of the variation) was correlated with expression differences between the glandular tissues (*R*^2^ = 0.88, *P *<* *0.001; Figure 2C), whereas the third (12.3% of the variation) and fourth principal components (9.2% of the variation), distinguished the epithelial tissues from one another (*R*^2^ = 0.85, *P *<* *0.001; Figure 2D and *R*^2^ = 0.69, *P *<* *0.001; Figure 2E, respectively).

**Figure 3:**
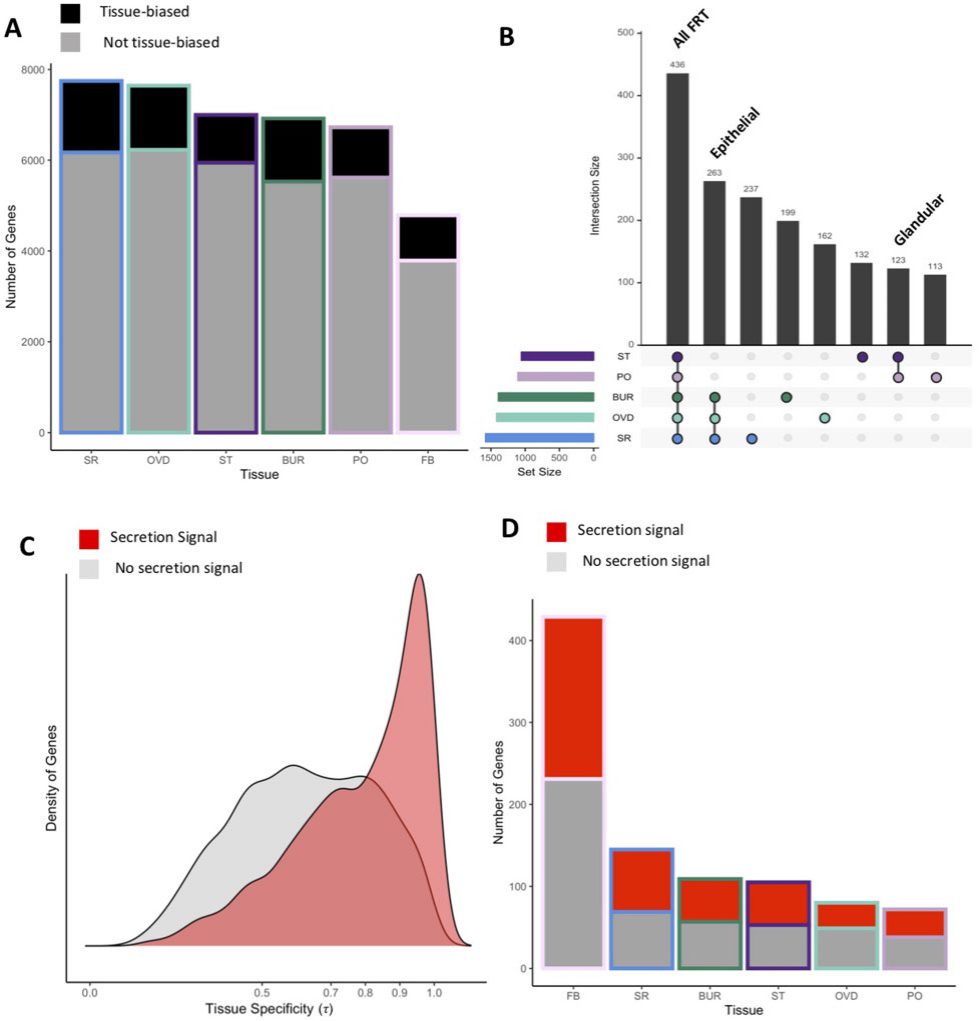
Identification of FRT-biased and tissue-specific genes. (A) The number of genes expressed in each tissue was significantly different and, in particular, the FRT-associated FB had the fewest number of expressed genes. The proportion of genes with biased expression in a particular tissue compared to the whole female (FDR < 0.001 and log2FC > 2) was also significantly different among tissues, with the lowest proportion in the glandular tissues the spermathecae (ST) and parovaria (PO) compared to the epithelial bursa (BUR), oviduct (OVD), and seminal receptacle (SR). (B) Intersections among genes with biased expression across FRT tissues (showing top 8 out of 31 intersections) identified large sets of genes with biased expression across multiple tissues. Specifically, we highlight the three largest intersections, genes with biased express in all FRT tissues, in all three epithelial tissues or in both glandular tissues. (C) Density distribution plot of genes expressed in the FRT as a function of tissue expression specificity (τ). We observed a significant difference in the distribution of τ between genes with (red) or without (gray) a secretion signal annotation. (D) We observed significant differences in the proportion of tissue-specific genes (τ > 0.9) across all tissues, with the greatest proportion and number in the FRT-associated fat body. Genes with tissue-specific expression were significantly enriched for secretion signal annotation (red) across all tissues.

To identify genes responsible for transcriptome variation across the FRT, we first identified those with biased expression in each tissue [*i.e.*, genes with higher expression in a tissue compared to the whole female using a stringent cutoff of FDR < 0.001 and log2-fold change (FC) > 2]. We found significant differences among tissues in both the number of genes expressed (χ^2^= 1364.0, *df* = 5, *P *=* *8.69^−293^) and the proportion of genes with tissue-biased expression (χ^2^= 120.9, *df* = 5, *P *=* *2.06e^−24^; Figure 3A). Specifically, epithelial tissues had the greatest number and proportion of tissue-biased genes. Biased expression using these criteria was not mutually exclusive among tissues, and we identified three large overlapping sets of genes with biased expression, including those with expression (1) in all FRT tissues, (2) restricted to epithelial tissues, or (3) restricted to glandular tissues (Figure 3B).

We next investigated the functional enrichments for genes with tissue-biased expression in all FRT tissues (*i.e.*, genes that were expressed at higher levels in every FRT tissue compared to the whole body minus the FRT). We found that genes with biased expression in all FRT tissues were significantly enriched for a UniProt secretion signal annotation (*i.e.*, genes whose protein products are likely to be either secreted or become membrane components) and had GO functional annotation enrichments related to immune response (Supplementary Table S2). These may represent functions to which all tissues contribute and are required in a spatially distributed fashion across the entirety of the FRT. The functional enrichments for genes with tissue-biased expression in epithelial or glandular tissues reflected the histological characteristics of these tissues (Supplementary Table S2). In particular, genes with epithelial tissue-biased expression were enriched for muscularization (*myofibril assembly*, *P *=* *2.2e^−5^; *sarcomere organization*, *P *=* *2.0e^−4^; *z-disc*, *P *=* *1.7e^−5^) (Nonidez 1920; Kapelnikov *et al.* 2008a; Heifetz and Rivlin 2010) and innervation (*negative regulation of neuromuscular synaptic transmission*, *P *=* *0.04) (Häsemeyer *et al.* 2009). Secretion signal annotation (*P *=* *4.3e^−6^) was also significantly enriched in all epithelial tissues, indicative of specific epithelial contributions to the extracellular environment and consistent with the observation of secretory activity in the oviduct and seminal receptacle (Kapelnikov *et al.* 2008a; Heifetz and Rivlin 2010). In contrast, genes with glandular tissue-biased expression had a significant enrichment relating to secretory pathways (*endomembrane system*, *P *=* *1.8e^−4^) which is consistent with the greater representation of secretory cells in these tissues. Interestingly, they did not exhibit an enrichment for secretion signal annotation (*P *=* *0.5) which indicates that the glandular tissues do not express a common repertoire of secreted proteins as would be expected if were primarily functionally redundant.

### Rapid evolution of tissue-specific genes encoding secreted proteins

We further explored the distinct transcriptional profiles of each tissue by estimating tissue-specificity of gene expression using τ (a measure from 0 to 1), where tissue specificity increases as τ approaches 1 (Yanai *et al.* 2005). We found that genes with secretion signal annotation had a distribution significantly biased toward higher values of τ relative to those that do not (*D* = 0.34, *P *<* *0.0001; Figure 3C). This observation was further supported by the significant enrichment for secretion signal annotation amongst genes with tissue-specific expression (τ ≥ 0.9; *P *<* *0.001 for all tissues; Figure 3D, Supplementary Figure S3). As such, each tissue expresses a largely unique set of secreted proteins which could generate regional variation in the extracellular environments across the FRT.

Tissue-specific GO enrichments corresponded to known characteristics and functions of FRT tissues and also identified potentially novel contributions. For example, tissues were enriched for the production of putatively secreted proteins that may function in chemical sensing (*odorant binding*, *P *=* *5.06e^−6^ in the bursa) or digestion of the transferred ejaculate (*serine-type endopeptidases*, *P *=* *8.88e^−15^ in the spermathecae; Supplementary Table S3). Interestingly, putatively secreted parovaria-specific genes were enriched for reproductive functions (*multicellular organism reproduction*, *P *=* *3.42e^−5^; Supplementary Table S3) due to the presence of genes previously characterized as SFPs. We note that as these genes are coexpressed in both the male accessory gland and FRT tissues, it is likely some SFPs are produced by both males and females. We also observed tissue-specific GO enrichments that may represent complementary functionality between tissues. For example, the sperm-storage organs (seminal receptacle and spermathecae) exhibited enrichments for distinct immune response mechanisms (*scavenger receptor activity*, *P *=* *0.007 in the seminal receptacle; *galactose binding*, *P *=* *0.002 and *calcium-dependent cell adhesion*, *P *=* *0.02 in the spermathecae; Supplementary Table S3). This observation is consistent with the hypothesis that different regions of the FRT have variable immune requirements (Kapelnikov *et al.* 2008b). Notably, the FRT-associated fat body had a significantly larger number of tissue-specific genes relative to FRT tissues, despite having a smaller number of expressed genes (χ^2^= 1085.30, *df* = 5, *P *=* *2.08e^−232^; see Supplementary results for further analysis of FRT-associated FB).

**Figure 4:**
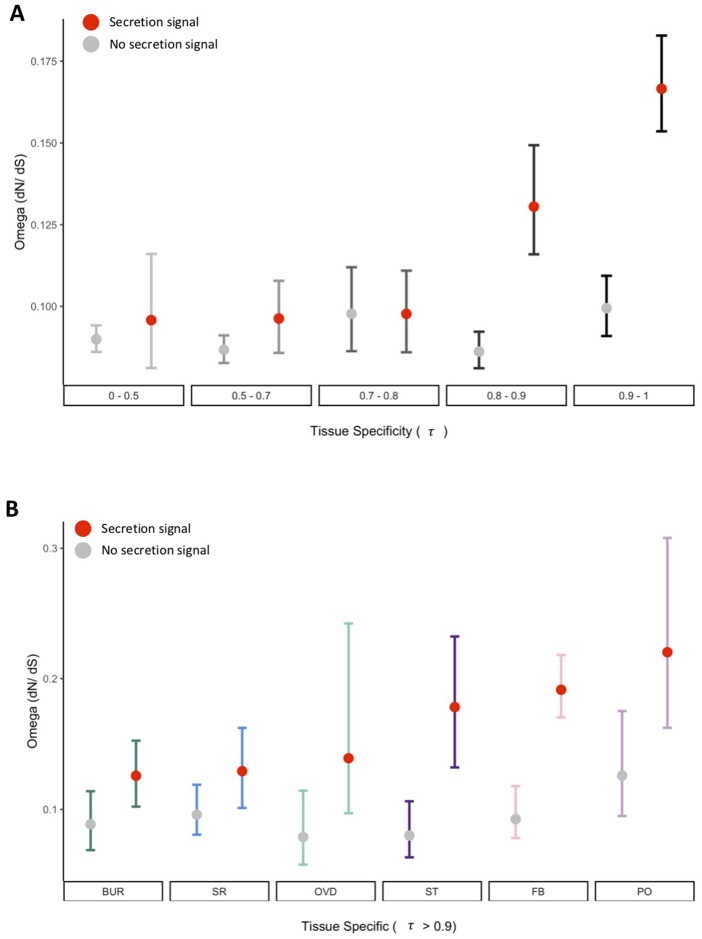
Molecular evolution of FRT expressed genes. (A) Evolutionary rate (ω; dN/dS) was significantly associated with tissue specific expression, secretion signal annotation, and their interaction. (B) Amongst tissue-specific genes (τ > 0.9), evolutionary rate was significantly associated with secretion signal annotation, and the interaction between secretion signal annotation and tissue. Error bars represent bootstrapped 95% confidence interval of the estimated mean.

We next tested the prediction that secreted FRT gene products evolve more rapidly than nonsecreted products as has previously been found, particularly in reproductive genes (Swanson *et al.* 2001, 2004; Liao *et al.* 2010). Evolutionary rates (dN/dS; ω) were estimated based on the molecular divergence of orthologs between *D. melanogaster* and *yakuba*. We found significant effects of tissue specificity (*H* = 85.4, *df* = 4, *P *=* *7.54e^−17^), secretion signal annotation (*H* = 140, *df* = 1, *P *=* *1.60e^−31^), and the interaction of both factors (*H* = 202.57, *df* = 9, *P *=* *5.75e^−38^) on the evolutionary rate of all FRT-expressed genes (Figure 4A). In particular, genes with higher tissue-specificity (τ categories of 0.8–0.9 and 0.9–1) and secretion signal annotation had a higher evolutionary rate than genes without a signal annotation (*P ≤ *5.2e^−7^ for all comparisons). However, we note that secretion signal sequences have been found to evolve under relaxed selection which may contribute to more rapid evolution of these genes (Williams *et al.* 2000; Li *et al.* 2009). Next, we analyzed tissue-specific genes (τ ≥ 0.9) and observed significant effects of secretion (*H* = 80.67, *df* = 1, *P *=* *1.60^−18^) and the interaction between tissue and secretion (*H* = 107.41, *df* = 11, *P *=* *3.6e^−17^) on evolutionary rate, although there was not a significant effect of tissue alone (*H* = 11.23, *df* = 5, *P *=* *0.28; Figure 4B). Notably, many of the rapidly evolving tissue-specific genes were X-linked genes or are also SFPs (Supplementary Table S1).

### Correlated, transient expression changes across the FRT in response to mating

**Figure 5:**
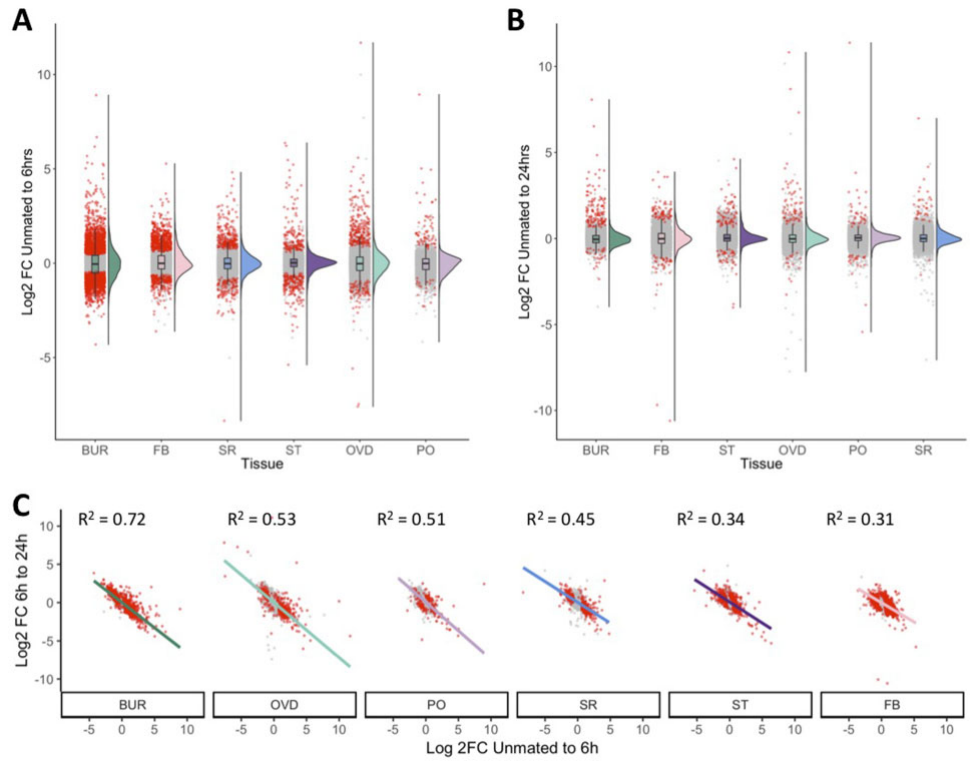
Postmating expression response across the FRT. (A) Dot-plot and density plot of log2FC in gene expression between unmated and 6 h postmating expression for each tissue. Tissues had significantly different numbers of differentially expressed genes and are ordered according to number of differentially expressed genes (red). All tissues [oviduct (OVD), seminal receptacle (SR), parovaria (PO), and fat body (FB)] except the bursa (BUR) and spermathecae (ST) had more genes upregulated than downregulated. (B) Dot-plot and density plot of log2FC in gene expression between unmated and 24 h postmating expression for each tissue. Tissues had significantly different numbers of differentially expressed genes and are ordered according to number of differentially expressed genes (red). All tissues, except the parovaria had more genes upregulated than downregulated. (C) Linear regression analysis of each tissue revealed a significant correlation between log2FC in expression from unmated-to-6 h postmating and 6 h -to-24 h postmating. Differentially expressed points at either 6 or 24 h postmating compared to unmated are indicated in red. Tissues are ordered by strength of correlation.

We next compared the temporal patterns of gene expression among FRT tissues by examining two postmating time points (6 and 24 h; Figure 5, A and B). We observed significant differences among tissues in the numbers of genes differentially expressed at each time point (relative to unmated tissues), with the greatest transcriptional response in the bursa at both time-points (6 h: χ^2^ = 5425.2, *df* = 5, *P *=* *0.00; 24 h: χ^2^ = 123.9, *df* = 5, *P *=* *4.67e^−25^). Despite these differences in the magnitude of response, we found that all tissues exhibited a significantly greater number of differentially expressed genes at 6 h (χ^2^ = 5.3, *df* = 1, *P *=* *0.02 for all tissues) and more genes upregulated than downregulated at both times postmating in the majority of tissues (average per tissue 6 h postmating 377 ± 159 upregulated and 283 ± 167 downregulated, χ^2^ ≤ 25.8, *df* = 1, *P ≤ *3.78e^−7^ for all except the bursa and spermathecae; average per tissue 24 h postmating 62 ± 13 upregulated and 22 ± 5 downregulated, χ^2^ ≤ 7.4, *df* = 1, *P ≤ *0.006 for all except the parovaria).

To investigate relationships in gene expression changes across the full experimental time-course, we examined the correlation in gene expression changes between unmated-to-6 h and 6–24 h within each tissue and found significant inverse correlations in all tissues (Figure 5C; average *R*^2^ = 0.48 ± 0.06; *P *<* *0.0001 for all). These relationships indicated that FRT tissues generally had a transient response to mating, in which the majority of gene expression changes at 6 h postmating (both up- and down-regulated) and returned to unmated-like levels by 24 h postmating. However, there was substantial variation across tissues. For example, the bursa showed the strongest inverse relationship in gene expression change between timepoints (*R*^2^ = 0.72), whereas the spermathecae (*R*^2^ = 0.34) and FRT-associated fat body (*R*^2^ = 0.31) exhibited weaker relationships. A complementary fuzzy clustering analysis, which identifies genes exhibiting common patterns of expression, produced similar results in all tissues (Supplementary Figure S4). Based on this common pattern of postmating response, we classified differentially expressed genes as either transient (*i.e.*, those that return to an unmated-like state by 24 h postmating) or persistent (*i.e.*, those that remain different from unmated at 24 h postmating). We found a greater proportion of transient genes in all tissues (79.0% ± 5.9% of all differentially expressed genes), although the proportion of transient genes varied significantly among tissues (χ^2^ = 317.2, *df* = 5, *P *=* *1.96e^−66^; maximum = 93.6% in the bursa, minimum = 55.0% in the parovaria).

We observed substantial functional coherence among transiently expressed genes (Supplementary Table S4). In particular, all epithelial tissues were enriched for the transient upregulation of genes related to translational activity (*nucleolus*, *P ≤* 4.06e^−10^ for all; *rRNA processing*, *P *≤ 0.001 for all epithelial tissues) and the bursa was also enriched for transcriptional activity (*DNA-directed RNA polymerase activity*, *P *=* *3.58e^−5^). A subset of tissues exhibited transient upregulation of immune response genes (*defense to Gram-positive bacteria*, *P *≤ 0.003 in the seminal receptacle and parovaria; *innate immune response*, *P *≤ 0.01 in the oviduct and parovaria). The parovaria was also enriched for transient upregulation of digestive enzymes (*proteolysis*, *P *=* *0.02). The majority of tissues exhibited a transient upregulation of secretion signal annotation genes (*P ≤* 6.55e^−4^ for all except bursa and FRT-associated fat body), suggesting postmating changes in tissue contributions to the extracellular reproductive environment. In contrast, there were minimal GO enrichments for transient downregulated genes, although several did also exhibit an enrichment of secretion signal annotation (*P *≤ 0.035 in the seminal receptacle, spermathecae and FRT-associated fat body; Supplementary Table S4). A minority of genes had persistent gene expression changes (21.0% ± 5.9% of all differentially expressed genes) and these exhibited less functional coherence than transient genes. One notable exception was the persistent upregulation of immune response genes in all epithelial tissues (*defense response to Gram-positive bacterium*, *P *≤ 0.02 in the bursa, oviduct and seminal receptacle; *innate immune response P ≤* 8.8e^−6^ in the bursa and oviduct).

**Figure 6:**
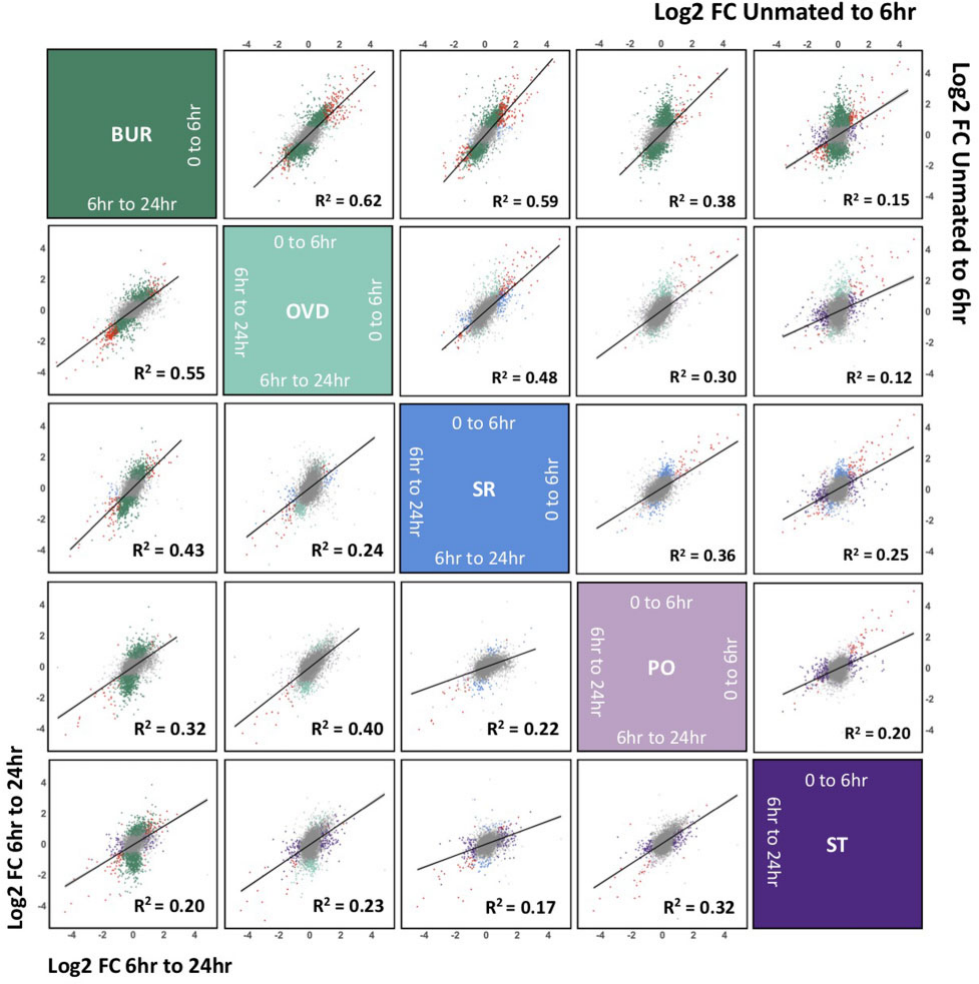
Correlated postmating expression changes across the FRT. Linear regression of log2FC in gene expression from unmated-to-6 h postmating between all tissues (top right of diagonal) and from 6 h -to-24 h postmating (bottom left of diagonal). Correlations were significant between all tissues at both time intervals. Genes significantly differentially expressed in both tissues are indicated red. Plots depicts genes within a range of four log2FC.

We predicted that the roles of FRT tissues in distinct postmating reproductive processes (*e.g.*, sperm storage, sperm ejection, ovulation, and mediating sperm competition) might be reflected in disparate postmating expression profiles. To test this prediction, we compared postmating gene expression changes in all pairwise combinations between FRT tissues and found significant correlations in all comparisons for both time-intervals (*P < *0.0001 for all; average *R*^2^ unmated-to-6 h = 0.35 ± 0.06; average *R*^2^ 6–24 h = 0.31 ± 0.04; Figure 6) although there was less similarity to the FRT-associated fat body (see supplemental results; Supplementary Figure S5). In addition, the majority of genes with significant postmating expression changes were differentially expressed in at least two tissues between unmated-to-6 h postmating (70.6% ± 11.6% upregulated and 51.4% ±11.5% downregulated) and 6–24 h postmating (51.7% ±9.1% upregulated and 63.6% ±10.4% downregulated). Notably, there were 16 genes significantly upregulated at 6 h postmating in all tissues, including four antimicrobial genes (*Atta*, *CecA1*, *Def*, and *Listericin*) and two serine endopeptidases (*Send2* and *CG17234*). Thus, despite the heterogeneity of transcriptome profiles across the FRT tissues, postmating responses were highly consistent and indicative of an integrated functional system.

## Discussion

The FRT, especially of species with internal fertilization, is a complex system of tissues that support fertility. Previous investigations, focused on a limited number of individual FRT tissues or genes, have identified regionally distinct expression patterns that may contribute to tissue-specific functions (Heifetz and Wolfner 2004; Allen and Spradling 2007; Kapelnikov *et al.* 2008a; Prokupek *et al.* 2008, 2009, 2010; Heifetz *et al.* 2014). In contrast developmental, morphological, and functional similarities among tissues, and particularly between the spermathecae and parovaria, support potentially concerted or redundant roles (Anderson 1945; Sun and Spradling 2012, 2013). Here, we advance our understanding of integrated functionality across the FRT through the systematic characterization of gene expression in individual tissues before and after mating.

Histological and ultrastructural evidence has shown that most FRT tissues have the secretory capacity necessary to contribute to the extracellular environment (Nonidez 1920; Kapelnikov *et al.* 2008a; Heifetz and Rivlin 2010; Sun and Spradling 2012). These secreted products likely contribute to the cascade of events leading to fertilization and oviposition, including interactions with sperm and SFPs. The formation of an FRT environment that can rapidly respond to mating (*i.e.*, a “poised” state) has been hypothesized to occur prior to mating during posteclosion maturation (Mack *et al.* 2006; Carmel *et al.* 2016). However, the contributions of specific tissues that form this environment have remained elusive. Using complementary approaches, we identified both an enrichment of genes encoding secreted proteins that may represent a core FRT secretome (i.e., expressed at high levels in all tissues), as well as tissue-specific genes encoding secreted proteins that may establish specialized FRT microenvironments.

Tissue-specific secreted gene products may result in microenvironments within the FRT or they may diffuse throughout the extracellular environment. For example, and consistent with previous studies (Allen and Spradling 2007; Prokupek *et al.* 2008), we found the majority of FRT-expressed serine endopeptidases had spermathecae-specific expression. It is possible these proteases have specific functions related to sperm storage in the spermathecae. Alternatively, they may be transported or diffuse to other tissues, such as the bursa, where they can participate in the degradation of the ejaculate as proposed in other *Drosophila* species (Kelleher and Pennington 2009), butterflies (Meslin *et al.* 2015), and mice (Li *et al.* 2017). Both hypotheses are supported by the analysis of FRT mutants without spermathecal secretory capabilities which demonstrate both local (*e.g.*, sperm movement into the spermathecae) and distant (*e.g.*, sperm survival in the seminal receptacle and regulation of ovulation and oviposition) effects of spermathecal secretions (Anderson 1945; Allen and Spradling 2007; Schnakenberg *et al.* 2011; Sun and Spradling 2012, 2013). The function of spermathecal secretory products in other tissues is further supported by a comparative analysis of 113 *Drosophila* species which showed that spermathecal secretory cells were consistently conserved, even if the evolutionary loss of sperm storage in the spermathecae resulted in a size reduction of the spermathecal capsule (Pitnick *et al.* 1999). Although dispersion mechanisms within the FRT are not well studied, it is possible that, similar to exosomes produced by male accessory glands (Corrigan *et al.* 2014), FRT tissues may also produce secretory vesicles for the targeted transport of molecular cargo.

Notably, tissue-specific genes with secretion signal annotations were the most rapidly evolving FRT-expressed genes. Tissue-specific expression profiles provide a more nuanced understanding of previously identified rapidly evolving FRT genes (Swanson *et al.* 2004; Panhuis and Swanson 2006; Prokupek *et al.* 2008, 2010). In addition, our observation of evolutionary rate heterogeneity across FRT tissues is similar to patterns previously observed across tissues of the male reproductive system (Dean *et al.* 2009). We postulate that the rapid evolution of genes with FRT specific expression is likely due to their accessibility within the reproductive environment and involvement in co-evolving interactions with sperm, SFPs, or microbes (Swanson and Vacquier 2002; Clark *et al.* 2006).

In response to male reproductive stimuli, the FRT rapidly transitions from an unmated to a mated state and commences a diverse repertoire of morphological and physiological transitions (Carmel *et al.* 2016). Consistent with previous analyses of the FRT as a single structure (Mack *et al.* 2006), we observed a transient peak of transcriptional changes approximately 6 h after mating that are greatly reduced by 24 h postmating. This pattern of transient response was especially prominent in the bursa, which also showed the greatest breadth of transcriptional response to mating, consistent with its’ diverse role in a variety of postmating functions from receipt of the ejaculate through to oviposition. Functional enrichments amongst transient upregulated genes were generally consistent with established postmating changes in the FRT. For example, a notable change in the bursa and oviduct was the upregulation of nervous system genes. This may be related to increased innervation following mating, including ovulin-dependent expansion of octopamine neurons (Rubinstein and Wolfner 2013), as well as regionally and temporally specific synaptic vesicle release (Heifetz and Wolfner 2004). The importance of nervous system activity in the FRT is demonstrated by the functions of epithelial tissue associated neurons in female receptivity, egg laying, and remating behaviors as well as sperm storage and sperm competition outcomes (Häsemeyer *et al.* 2009; Chow *et al.* 2013).

We also observed an increase in immune gene expression across FRT tissues, consistent with previous observations that stimulation of the immune system is a predominant characteristic of female postmating responses (Lawniczak and Begun 2004; McGraw *et al.* 2004; Peng *et al.* 2005b; Kapelnikov *et al.* 2008b). However, the extent of increases in immune gene expression varied across tissues, and persistent upregulation of immune response was only observed in the epithelial tissues. The specificity of postmating immune response is also intertwined with variable levels of immune gene expression across tissues suggesting regional specificity of immune potentiation across the FRT, as previously observed in the oviduct (Kapelnikov *et al.* 2008b). We postulate that the heterogenous immunity landscape may be, in part, due to the distinct functional requirements of tissues, such as those involved in prolonged interactions with male-derived substances (*i.e.*, those involved in sperm storage) (Orr and Brennan 2015). Reproductive immunity is often assumed to protect against sexually transmitted pathogens or be an immunogenic response to the sperm-cells of males (Arnqvist and Rowe 2005; Wigby *et al.* 2019). In addition to these functions, there is increasing evidence that the postmating immune response may govern mechanisms related to postmating sexual selection (Birkhead *et al.* 1993; Morrow and Innocenti 2012; Wigby *et al.* 2019).

The coordinated postmating response across the FRT is consistent with highly integrated functions across tissues mediating the physiological changes required to support fertility. This coordination may arise due to a combination of nonmutually exclusive male and female mechanisms. First, the initial stimuli are likely to derive from ejaculate components (*i.e.*, SFPs and sperm proteins), which are known to mediate a wide array of female postmating responses (Wolfner 2002). A well-studied example of this is the SFP sex-peptide (SP), which is distributed across the FRT both freely and attached to sperm (Peng *et al.* 2005a), and induces postmating changes through interactions with its female-expressed receptor, sex peptide receptor (SPR) (Yapici *et al.* 2008). Notably, we found that SPR, as well as female genes in the SP network, were expressed in all FRT tissues (Yapici *et al.* 2008; Findlay *et al.* 2014). In addition to SFPs, other ejaculate cargo such as contents of extracellular vesicles (Corrigan *et al.* 2014) could initiate coordinated responses. Second, there are likely to be female-mediated mechanisms that govern the rapid response and coordination within and between FRT tissues. This may include microRNAs (miRNAs) which have mating responsive expression profiles and influence postmating changes in female receptivity, egg-laying, and gene expression (Fricke *et al.* 2014; Zhou *et al.* 2014; Carmel *et al.* 2016). Mating has also been shown to induce regionally specific neuromodulator release across the FRT, which could initiate signaling cascades across tissues and influence ovulation, sperm storage and oviposition (Rubinstein and Wolfner 2013; Heifetz *et al.* 2014; Rezával *et al.* 2014). Thus, signaling molecules from both the male and female may regulate co-ordinated system-wide responses to mating.

It is noteworthy that persistent expression changes tended to be more distinct among tissues relative to transient postmating responses. These differences may reflect regionally specific changes necessary to support sustained and spatially restricted functions. The spermathecae, in particular, has consistently been an outlier in patterns of postmating changes relative to other FRT tissues (Prokupek *et al.* 2009), and our results suggest that this pattern is largely due to the greater extent of persistent gene expression changes in this tissue. This interpretation is consistent with the unique functions of the spermathecae relating to sperm survival in storage (Anderson 1945; Allen and Spradling 2007; Schnakenberg *et al.* 2011). Thus, tissues and gene products involved in prolonged interactions with ejaculate components, and in particular proteins in or bound to sperm, appear to be more likely to experience persistent expression changes.

This comprehensive study of gene expression across FRT tissues emphasizes the importance of molecular investigations that isolate component parts of complex biological systems. Establishing the molecular relationships among tissues has provided new insights into the formation of the extracellular FRT environment, including rapidly evolving products that may be involved in ejaculate interactions, and the coordination of FRT responses to mating. However, a more complete characterization of the FRT environment will depend on the generation of high resolution quantitative proteomic data over a fine-scale postmating time course as well as analyses of metabolites, ionic concentrations, and additional biochemical characteristics. Moreover, comparative analyses within a phylogenetic framework are needed to understand the selection pressures influencing FRT evolution. This study provides the foundation for future investigation across evolutionary informative taxa using complementary “omic” approaches and targeted genetic manipulations to precisely establish FRT functionality.
